# Poly-l-lactic acid microspheres delay aging of epidermal stem cells in rat skin

**DOI:** 10.3389/fimmu.2024.1394530

**Published:** 2024-05-31

**Authors:** Yunxian Dong, Youliang Zhang, Hao Yu, Lingcong Zhou, Yaan Zhang, Haibin Wang, Zhicheng Hu, Shengkang Luo

**Affiliations:** ^1^ Department of Plastic and Cosmetic Surgery, Nanfang Hospital, Southern Medical University, Guangzhou, China; ^2^ Department of Plastic and Reconstructive Surgery, Guangdong Second Provincial General Hospital, Guangzhou, China; ^3^ Department of Burns, Wound Repair and Reconstruction, the First Affiliated Hospital of Sun Yat-sen University, Guangzhou, China

**Keywords:** poly-l-lactic acid, epidermal stem cells, skin aging, anti-aging, mesotherapy

## Abstract

**Objective:**

Injectable skin fillers offer a wider range of options for cutaneous anti-aging and facial rejuvenation. PLLA microspheres are increasingly favored as degradable and long-lasting fillers. The present study focused solely on the effect of PLLA on dermal collagen, without investigating its impact on the epidermis. In this study, we investigated the effects of PLLA microspheres on epidermal stem cells (EpiSCs).

**Methods:**

Different concentrations of PLLA microspheres on epidermal stem cells (EpiSCs) *in vitro* through culture, and identification of primary rat EpiSCs. CCK-8 detection, apoptosis staining, flow cytometry, Transwell assay, wound healing assay, q-PCR analysis, and immunofluorescence staining were used to detect the effects of PLLA on EpiSCs. Furthermore, we observed the effect on the epidermis by injecting PLLA into the dermis of the rat skin *in vivo*.

**Results:**

PLLA microspheres promote cell proliferation and migration while delaying cell senescence and maintaining its stemness. In vitro, Intradermal injection of PLLA microspheres in the rat back skin resulted in delayed aging, as evidenced by histological and immunohistochemical staining of the skin at 2, 4, and 12 weeks of follow-up.

**Conclusion:**

This study showed the positive effects of PLLA on rat epidermis and EpiSCs, while providing novel insights into the anti-aging mechanism of PLLA.

## 1 Introduction

Cutaneous aging, a natural and inevitable process of the body, means a reduction in the number and functionality of epidermal cells and fibroblasts, as well as diminished synthesis and atrophy of elastin and collagen (1). The physiological manifestations of skin aging include thinning, atrophy, dryness, progressively prominent wrinkles, loss of elasticity, accompanied by pruritus, irregular pigmentation (2). These changes have increasingly impacted individuals’ pursuit of beauty, particularly in regards to the growing emphasis on facial skin aging. Consequently, strategies for delaying the aging process and achieving facial rejuvenation have become areas of substantial interest within both the beauty and fashion industry as well as among plastic surgeons (3, 4).

Epidermis aging plays a pivotal role in age-related alterations of the skin, as it is crucial for maintaining skin health and resilience against external environmental stressors (5, 6). The epidermis primarily consists of keratinocytes and basal cells, which are instrumental in upholding skin homeostasis and safeguarding against physical, chemical, and infectious damage (7, 8). The epidermal stem cells (EpiSCs), which are located in the basal cell layer, serve as the “seed” cells for self-renewal of the epidermis (9, 10). During the healing process of skin wounds, EpiSCs also have demonstrated a powerful ability to repair injury (11, 12). At the single-cell level, COL17A1^Hi^ basal cells in homeostatic skin can promote basal-spindle cell differentiation and wound healing after activation (13). EGFR-mediated EpiSC motility drives skin regeneration through COL17A1 proteolysis, which provides a new therapeutic method for age-related regeneration of damaged skin (14). A decrease in the number of EpiSCs may lead to reduced differentiation of keratinocytes, resulting in a thinning of the skin (15). The basement membrane band in young skin exhibits waviness, whereas it becomes flat in aged skin, leading to compromised structural integrity and mechanical stability (16).

Filler injection currently serves as the primary approach for facial rejuvenation (17–19). The most commonly employed degradable dermal fillers at present encompass crosslinked hyaluronic acid (HA) and collagen-stimulating fillers (20). The collagen-stimulating fillers, such as Poly-L-Lactic Acid (PLLA), which have a duration of effectiveness for up to 2 years, is highly favored by individuals and has been widely clinical utilized (21, 22). Mechanically, recent study demonstrated that PLLA could induce fibroblasts to produce collagen by continuously inflammatory response and promoting sustained activation of M2 macrophage polarization (23). The supplementary or regenerated collagen can effectively tighten the skin and diminish wrinkles, thus exerting an anti-aging effect (24, 25).

Mesotherapy is an intradermal injection of filler that is believed to enhance skin radiance and vibrancy (26, 27). However, traditional PLLA injections usually target the subdermis rather than the mesoderm. Current studies have predominantly focused on the effects of PLLA on the dermis, with limited exploration of its impact on the epidermal layer, particularly EpiSCs (28). In this study, we investigated the impact of PLLA on the *in vitro* biological functionality of rat EpiSCs and evaluated the effects of intradermal injection of PLLA on skin aging in rats.

## 2 Methods

### 2.1 Degradation of PLLA *in vitro*

Firstly, PLLA (Loviselle, SinoBiom, Changchun, China; Specification: 340 mg/5 mL) were placed in 1% phosphate buffered saline (PBS, 10010023, Gibco) for 10, 20 and 30 days, and then the morphological changes of the microspheres were observed under an optical light microscope. In addition, the diameter of the microspheres were also statistically measured. Since EpiSCs were used in subsequent experiments, the changes of PLLA microspheres in the EpiSCs specialized medium (keratinocyte serum-free medium, K-SFM, 17005042; Gibco) were also examined.

### 2.2 Isolation and culture of rat EpiSCs

The back skin of the Sprague–Dawley (SD) rats (six-week-old) were was excised and placed in a 15ml centrifuge tube containing 1% PBS. The fascia layer was excised, leaving only the epidermis and dermis intact. The skin was then cut into 1 × 1 cm^2^ sections and subjected to digestion in 2 ml of × 10 Tryple (A1217702; Gibco) at 37°C for a duration of 30 min. Following digestion, the skin tissue was carefully separated using sterile blades and tweezers to isolate the epidermis from the dermis.

For EpiSCs, the isolated epidermal tissue was collected and immersed in a 0.25% pancreatic enzyme solution at 37°C for a brief period of digestion lasting approximately 5 minutes. Subsequently, it was gently swirled, shaken, filtered through a 70 μm cell filter at a speed of 1500 rpm, and centrifuged for another five minutes to obtain cell precipitate consisting primarily of epidermal basal cells. These primary representative skin stem cells were then resuspended in K-SFM before being inoculated into fibronectin (FN, 5 μg/cm^2^, Shanghai Fibronectin Biotechnology, China) -coated culture bottles. After incubation at 37°C for two hours, the adherent cells were identified as primary EpiSCs when changing the medium.

For Fibroblasts, the isolated dermal tissue collected and digested with collagenase type II (S10054, 0.8 mg/ml, Yuanye, China) at 37°C for 6h. Then, after the residual tissue was filtered, the obtained cell suspension was centrifuged, re-suspended using the medium, and transferred to a petri dish for further culture.

### 2.3 Immunofluorescence staining

EpiSCs were cultured with different treatments in confocal glass bottom for 48 h. Then, the cells were fixed with 4% paraformaldehyde for 20 min, infiltrated with 0.25% Triton-×100 for 20 min, sealed with 0.5% goat serum at 37°C for 1 h, and then incubated at 4°C overnight with primary antibody. After being washed, the bound antibodies were reacted with fluorescent secondary antibodies at room temperature for 1 h and co-stained with DAPI for 15 min. The fluorescent signals were captured under a CLSM (LSM880 Basic Operation, Zeiss). The primary antibodies included anti-Integrin-α6 (1:200, ab235905, Abcam), anti-Cytokeratin 19 (CK19, 1:400, ab84632, Abcam), anti-Cytokeratin 15 (CK15, 1:400, ab80522, Abcam), anti-CD71 (1:400, ab22391, Abcam), anti-CD31 (1:400, ab24590, Abcam), anti-CD34 (1:400, ab81289, Abcam). The second antibodies included Goat Anti-Mouse IgG (1:500, SA00009–1, Proteintech), Goat Anti-Rabbit IgG (1:500, SA00009–2, Proteintech). For identification of EpiSCs, the cells at passage 2 were used.

### 2.4 Flow cytometry

For identification of EpiSCs, the collected third-generation cells were PBS and then were fixed and permeabilized using the BD Cytofix/Cytoperm™ Fixation/Permeabilization Kit. Incubation buffer (3ml) was added to the cells and centrifuged after washing. In each tube, the cells were suspended with 100 μL incubation buffer and blocked at room temperature for 10 min. Next, the primary antibody was incubated at room temperature for 1 h. Cells were then incubated with fluorescently labeled secondary antibodies at room temperature for 30 min. The cells were then centrifugally rinsed in incubation buffer and finally analyzed by flow cytometry. The primary antibodies are as follows: p63, Abcam, ab124762, 1:200; α6-integrin, Abcam, ab77906, 1:200.

For the detection of apoptosis, EpiSCs were seeded in 6-well plates and cultured overnight. PLLA microspheres was added to the final concentrations of 0, 250, 500, 2000 µg/mL in each well and cells were incubated in incubator for 6 hours. The EpiSCs were cultured at 37°C for another 24 hours. After washing, they were harvested and stained with Annexin V-FITC and propidium iodide (PI, 4abio, China) in the dark. Subsequently, the stained cells were subjected to examination using flow cytometer.

### 2.5 Cell Counting Kit-8 assay

EpiSCs (3×10^3^ cells/well) were cultured in 96-well plates overnight and treated in triplicate with different doses of PLLA (0, 50, 100, 250, 500, 750, 1000 and 2000 μg/mL). After incubating the culture for 24–48 hours, cell viability was determined using a CCK8 kit (Fudebio, Hangzhou, China) per the manufacturer’s protocol.

### 2.6 Cell live/dead staining

EpiSCs were cultured in 6-well plates and when they reached 90% confluence, PLLA (0, 250, 500, 2000 μg/mL) were added. After incubation for 48 hours, cells were washed 3 times with PBS, then incubated with Calcein-AM/propyl iodide (PI) for 30 minutes, and observed under fluorescence microscope.

### 2.7 Transwell assay

**Figure 1:**
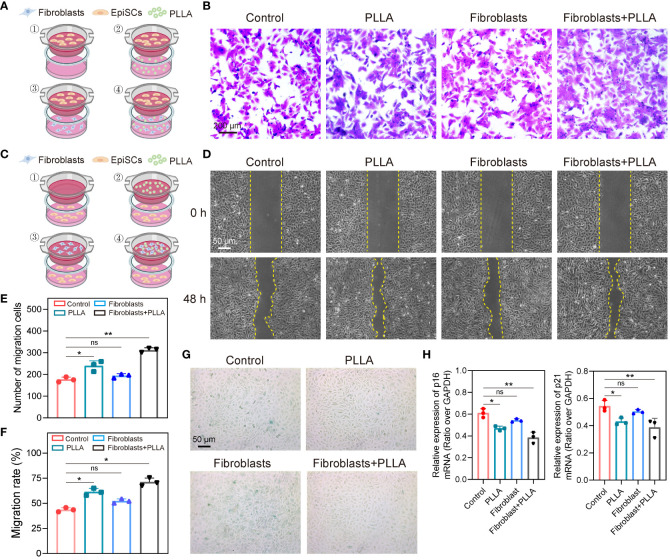
Effects of PLLA microspheres on migration and senescence of epidermal stem cells. **(A)** Schematic diagram, **(B)** images and **(E)** statistical analysis of Transwell migration assay of EpiSCs with different treatments. **(C)** Schematic diagram and **(D)** images of wound healing results of EpiSCs in different groups and **(F)** its statistical results. **(G)** β-Galactosidase (β-Gal) staining images of different groups. **(H)** qPCR analysis of mRNA expression levels of p16 and p21 in different treatment groups. Data are presented as mean ± SD. * indicates p < 0.05; ** indicates p < 0.01; ns means no significance.

EpiSCs were centrifuged and resuspended in a serum-free medium. A suspension of 200 μL cells (approximately 5000 cells) was added to the transwell chamber (without gel), followed by the addition of 600 μL of 20% FBS medium to the lower cavity of the 24-well plate. After 24 h, the transwell chamber was removed and any unmigrated cells were eliminated. Subsequently, the cells were fixed with paraformaldehyde for a duration of 20 minutes and stained with a solution containing 0.1% crystal violet for an additional period of 30 minutes. Finally, photographs and cell counts were taken under a microscope. As shown in **Figure 1A**, epidermal stem cells were planted in the upper well, while different treatments were given at the bottom of the culture dish: ① Control group; ②PLLA group; ③ Fibroblast group; ④ Fibroblast +PLLA group. The cells were treated as mentioned above continuously from generation 3 to generation 6.

### 2.8 Wound healing assay

EpiSCs was cultured, and when they reached 90% compatibility, the monolayer cells were scratched with the tip of a straw. After washing out the non-adherent cells with PBS, the remaining cells were cultured for 48 h. The wound healing of cell monolayer in each well was photoimaged and the percent of wound healing was calculated using ImageJ software. For this section (**Figure 1C**), the bottom of the culture dish was covered with EpiSCs, and different treatments were given in the upper well: (1) Control group; ②PLLA group; ③ Fibroblast group; ④ Fibroblast +PLLA group. The cells were treated as mentioned above continuously from generation 3 to generation 6.

### 2.9 β-Galactosidase activity assay

EpiSCs were given different treatments and cultured in 6-well plates for 24 hours. Then, according to the procedure of β-galactosidase staining kit (G1580, Solarbio), the cells were fixed and stained and incubated overnight. Next, cells were photographed under a light microscope. The cells were treated as mentioned in “Wound healing assay” continuously from generation 3 to generation 6.

### 2.10 Real-time quantitative PCR

Total RNAs were extracted from EpiSCs using RNA Easy Fast Tissue/Cell kit (TIANGEN, China) and reversely transcribed into cDNA using the Thermo Scientific RevertAid First Strand cDNA Synthesis Kit (Thermo Scientific, Waltham, MA), according to the manufacturer s instructions. The relative levels of interesting gene mRNA transcripts to the control GAPDH were quantified by RT-qPCR using specific primers and SYBR Green PCR master mix (Toyobo, Osaka, Japan) on a Bio-Rad CFX96 Real Time PCR system (Bio-Rad, Hercules, USA). The cells were treated as mentioned in “Wound healing assay” continuously from generation 3 to generation 6. The sequences of primers are as follows (29, 30):

P16- Forward: 5’-TCCTTGGCTTCACTTCTGGCAAC-3’

P16-Reverse: 5’-TCCTTGGCTTCACTTCTGGCAAC-3’

P21- Forward: 5’-GAAAACGGAGGCAGACCAG-3’

P21-Reverse: 5’-TTCAGGGCTTTCTCTTGCAG-3’

Integrin-α6- Forward: 5′-GTCACCTTTGACACCCCAGATC-3′

Integrin-α6-Reverse: 5′-CAACTGTACCTCCAAAATACACC-3′

GAPDH- Forward: 5’-GACATGCCGCCTGGAGAAAC-3’

GAPDH -Reverse: 5’-AGCCCAGGATGCCCTTTAGT-3’

### 2.11 Animals and molding

**Figure 2:**
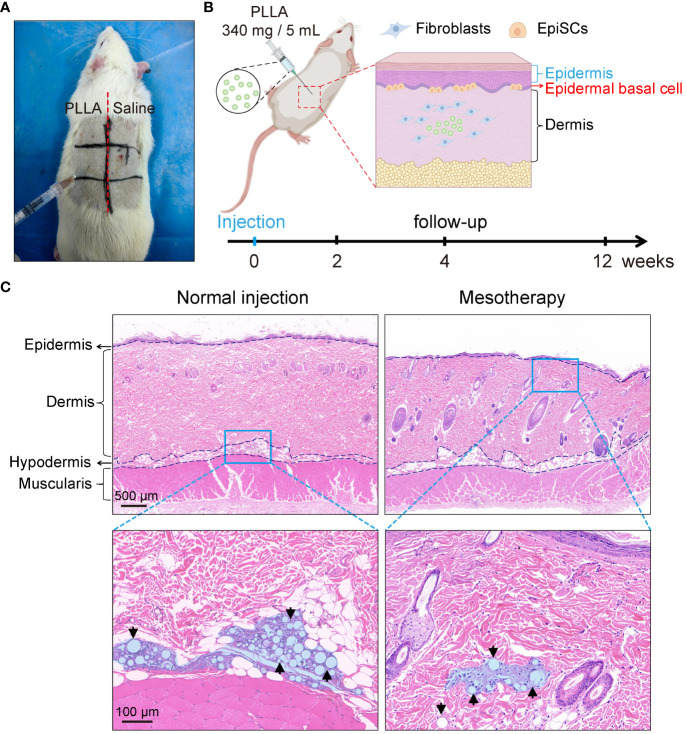
Mesodermal injection of PLLA in rats. **(A)** Photos of SD rats injected with PLLA. **(B)** Schematic diagram of mesoderm injection and animal experiments. **(C)** HE staining images of rat skin after subdermal injection (normal injection) and intradermal injection (mesotherapy) of PLLA microspheres.

Sixteen SD rats (male, six-week-old, weighing 280–300 g) were randomly divided into 2 groups. Rats were anesthetized intraperitoneally with 2% sodium pentobarbital (0.25 mL/100 g). After the hair was removed from the back of the rat, the labeled zones, as shown in **Figures 2A** and **B**, were injected with PLLA in the three zones on the left and saline on the right. Four points were injected into each zone, and 0.1 mL PLLA or saline was used at each point. The rats in each group were killed at the 0, 2, 4 and 12 weeks, respectively, and the skin tissues were harvested and stained. In this study, the injection depth was within the dermis, that is, the mesoderm. And it is different from conventional subdermal injection.

### 2.12 Histological assay

The dissected ear tissues were fixed in 10% of formalin overnight and paraffin-embedded. The tissue sections (5 µm) were regularly stained with H&E staining and Immunohistochemical (IHC) staining. The stained tissue sections were photoimaged and observed under a light microscope (Digital pathology section scanner, KFBIO, China, KF-PRO-020-HI). The primary antibodies of IHC included anti-CK19 (1:400, ab76539, Abcam). The ready-to-use type second antibody was HRP labeled goat anti-mouse secondary antibody (PR30012, Proteintech).

### 2.13 Statistical analysis

The difference between the two groups was analyzed by the unpaired t-test analysis and the difference among multiple groups was analyzed by one-way ANOVA and *post hoc* Bonferroni’s correction using SPSS 24.0 software. A P-value of < 0.05 was considered statistically significant.

## 3 Results

### 3.1 Release of PLLA microspheres

**Figure 3:**
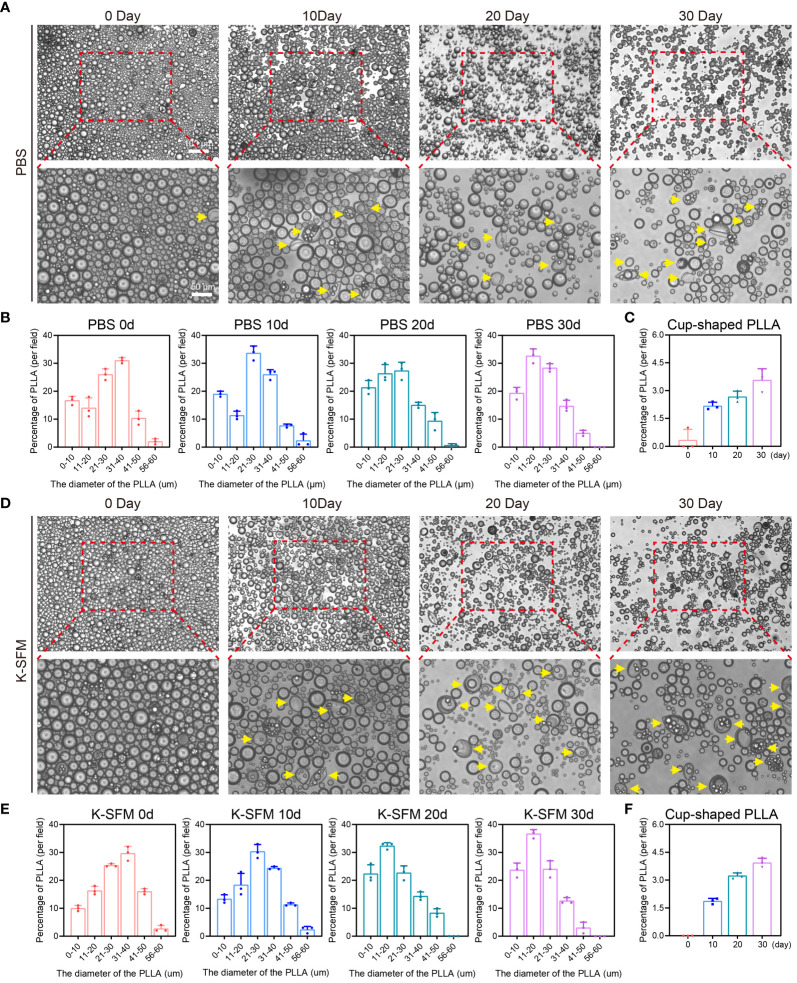
Degradation of PLLA microspheres in PBS and K-SFM. **(A)** Optical microscope photographsand **(B)** statistical analysis of the proportion of diameter distribution of PLLA microspheres at day 0, 10,20, and 30 in PBS. **(C)** Statistical analysis of the proportion of cup-shaped microspheres in PBS. **(D)** Optical microscope photographs and **(E)** statistical analysis of the proportion of diameter distribution of PLLA microspheres at day 0, 10,20, and 30 in K-SFM. **(F)** Statistical analysis of the proportion of cup-shaped PLLA microspheres in K-SFM. Yellow arrows: cup-shaped PLLA.

The release of PLLA microspheres *in vitro* was observed initially. The microspheres, when placed in PBS on days 10, 20, and 30, retained their spherical shape predominantly (**Figures 3A, D**). Then, the diameter of PLLA microspheres was measured revealing that the percentage of microspheres with a diameter ranging from 31–40μm decreased from 31.0% to 14.7%, while the proportion of microspheres with a diameter less than 20μm increased from 30.7% to 52.0% (**Figure 3B**). The aforementioned findings suggest a gradual reduction in the diameter of the PLLA microsphere over time, potentially indicating a slow degradation process similar to the previously reported mode of sustained release. Further, the release of PLLA microspheres in K-SFM was also measured, with results similar to those in the PBS group (**Figures 3D, E**). However, PLLA microspheres in K-SFM showed a faster degradation rate.

Additionally, a portion of PLLA microspheres exhibited a concave or cup-shaped morphology over time, and the proportion of cupped gradually increased (**Figures 3C, F**). Those findings imply that apart from exhibiting sustained release properties, PLLA microspheres may also undergo degradation through the occurrence of cracks (31).

### 3.2 Characterization and identification of EpiSCs

**Figure 4:**
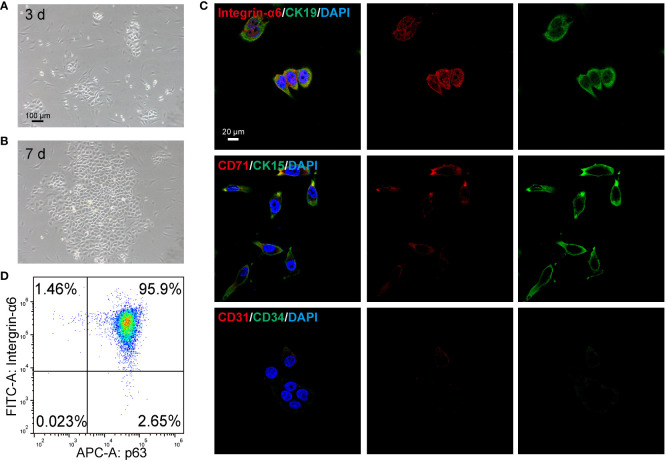
Identification of epidermal stem cells (EpiSCs) derived from rat skin. Light microscope photographs of the growth of EpiSCs on **(A)** day 3 and **(B)** day 7 after extraction. **(C)** Fluorescent confocal microscopic images of characteristic positive (Intergrin-α6, CK19, CK15, CD71) and negative markers (CD31, CD34) of EpiSCs. **(D)** Flow cytometry results of p63 and Intergrin-α6 in EpiSCs.

The method described in our previous study has demonstrated successful isolation of rat EpiSCs (32, 33). Then, the isolated epidermal stem cells were characterized. As shown in **Figure 4A**, following a 3-day culture of primary EpiSCs in K-SFM medium, the cells showed a pebble-shaped morphology and large nucleus under optical microscopy. By day 7, the EpiSCs displayed an arrangement resembling paving stones with tight intercellular connections (**Figure 4B**). The currently recognized markers for the identification of EpiSCs include Integrin-α6, CK15, CK19, and p63 (34–36). After the extracted EpiSCs were cultured to the third generation, immunofluorescence and flow cytometry were used to identify the specific marker and purity of cells. The immunofluorescence analysis revealed a high expression of basal cell membrane-related markers, including intergrain-α6, CK15, CK19, CD71 in EpiSCs (**Figure 4C**). Additionally, the absence of CD31 and CD34 staining in the negative control confirmed that the isolated cells were not vascular endothelial cells (**Figure 4C**). The flow cytometry results revealed that the population of intergrain-α6 and p63-double positive cells exhibited a remarkable purity level of 95.59% (**Figure 4D**). The above data validate the successful isolation of EpiSCs.

### 3.3 PLLA enhances the proliferation of EpiSCs

**Figure 5:**
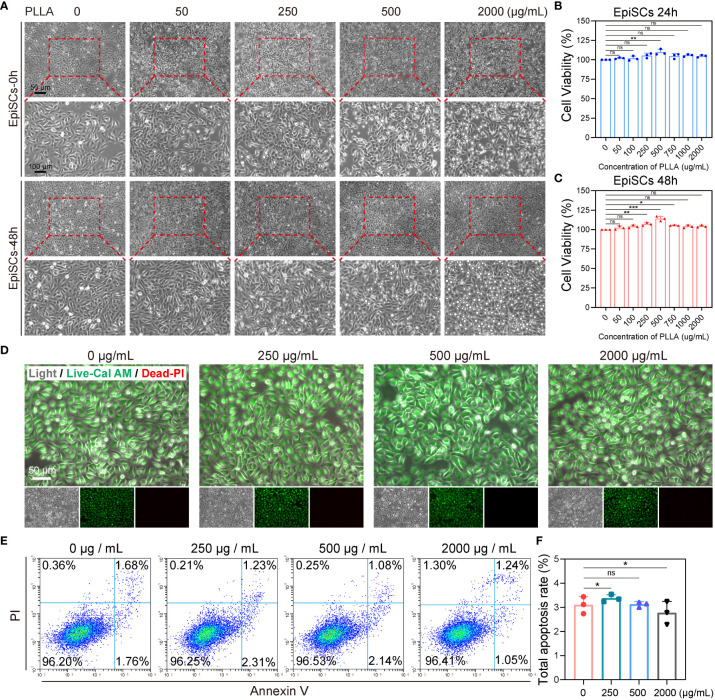
Effect of PLLA microspheres on viability and apoptosis of EpiSCs. **(A)** Photoscopic images of EpiSCs treated with 0–2000 μg/mL PLLA microspheres for 24 hours and 48 hours. Cell viability of EpiSCs was assessed using the CCK8 assay after treatment with PLLA microspheres at different concentrations for 24 h **(B)** and 48 h **(C)**. **(D)** Fluorescent images of epidermal stem cells in different treatment groups (Green for Live, Red for Dead). **(E)** Flow cytometry images and **(F)** statistical results of apoptotic EpiSCs treated with different concentrations of PLLA microspheres. Data are presented as mean ± SD. *indicates p < 0.05, **indicates p < 0.01, *** indicates p < 0.001; ns means no significance.

To investigate the impact of PLLA on EpiSCs, we first assessed the effects of various concentrations of PLLA (0, 50, 100, 250, 500, 750, 1000, and 2000 μg/mL) on cell viability. As depicted in **Figure 5A**, EpiSCs were exposed to PLLA microspheres at varying concentrations for 24 and 48 hours, resulting in favorable cellular growth. The analysis of cell viability using CCK8 demonstrated that only 500μg/mL of PLLA exerted a significant impact on cell viability after 24 hours (**Figure 5B**). After 48 hours, the cell viability at concentrations of both 250μg/mL and 500μg/mL reached levels of 116.7% and 126.3%, respectively, compared to the control group (0 μg/mL) with a viability rate of 100% (**Figure 5C**).

Furthermore, Live and Dead staining was conducted on the cells using Calcein-AM (green fluorescence indicating live) and PI (red fluorescence indicating death). It was observed that PLLA concentrations of 0, 250, 500, and 2000g/mL did not induce cell death as evidenced by minimal red fluorescence (**Figure 5D**). Then, apoptotic flow cytometry analysis revealed comparable apoptosis rates among the different treatment groups (**Figure 5E**): 3.10% (0 μg/mL), 3.37% (250 μg/mL), 3.12% (500 μg/mL), and 2.77% (2000 μg/mL). No significant differences were observed between these groups (**Figure 5F**). Above, we found that PLLA played a potential role in facilitating cellular proliferation, and subsequently determined 500 μg/mL as the optimal concentration for subsequent experiments.

### 3.4 PLLA slows the aging of EpiSCs

The anti-aging effects of PLLA microspheres on the skin have been previously reported. We investigated whether PLLA microspheres had an impact on senescence in rat EpiSCs. Normally, EpiSCs *in vitro* maintain good stemness until the 3rd generation, but exhibit signs of senescence and terminal differentiation towards keratinocytes by the 6th generation. Therefore, for this experiment, we utilized the 6th generation of EpiSCs. Stem cells are known for their strong proliferation capacity; hence, we assessed the proliferation and migration abilities of EpiSCs through Transwell and wound healing assay. To simulate the effect of intradermal injection of PLLA on the epidermal layer *in vivo*, we established an *in vitro* co-culture system consisting of epidermal stem cells and fibroblasts (**Figures 1A, C**).

The migration ability of EpiSCs was enhanced by PLLA, with a more pronounced effect observed in the fibroblast + PLLA group (**Figures 1B, E**). No difference was observed in the co-culture of EpiSCs and fibroblasts compared to the control group (**Figures 1B, E**). Additionally, PLLA promoted both proliferation and migration of epidermal EpiSCs in wound healing assay (**Figures 1D, F**). The presence of fibroblasts further enhanced the proliferative ability induced by PLLA (**Figures 1D, F**). Moreover, β-GAL (a senescence indicator) staining revealed a deeper blue-green color in the 6th generation EpiSCs, while addition of PLLA delayed cell senescence (**Figure 1G**). In **Figure 1H**, mRNA levels of p16 and p21 (cellular senescence markers) were also reduced by PLLA treatment. Collectively, these results indicate that PLLA promotes proliferation and delays senescence phenotype in EpiSCs with involvement from fibroblasts.

### 3.5 PLLA decreases the differentiation of EpiSCs

**Figure 6:**
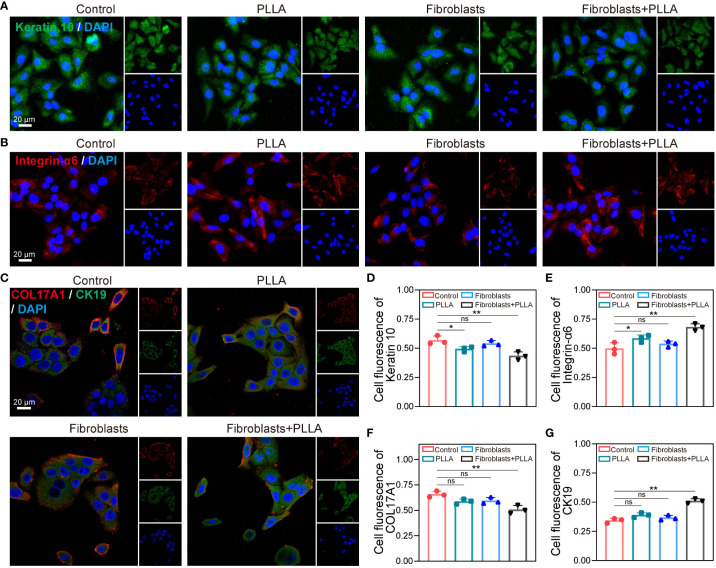
Impact of PLLA microspheres on the process of differentiation in EpiSCs. Representative fluorescence images of **(A)** Keratin 10, **(B)** integrin-α6, **(C)** COL17A1 and CK19 with different treatments. **(D-G)** Quantitative analysis results of fluorescence intensity. Data are presented as mean ± SD. *indicates p < 0.05, **indicates p < 0.01; ns means no significance.

In **Figure 6A**, EpiSCs cultured up to the 6th generation *in vitro* exhibited a pronounced expression of keratin 10, indicating their terminal differentiation into keratinocytes following mitosis. However, the expression of keratin 10 was comparatively lower in PLLA+ fibroblasts group (**Figure 6D**). Integrin-α6 serves as a crucial protein for EpiSCs’ adherence to the basement membrane and acts as an important marker for their stemness. We observed that the untreated cells displayed significantly diminished levels of integrin-α6 compared to the PLLA group (**Figures 6B, E**), suggesting that PLLA mitigates cell differentiation while preserving stemness. Recent studies have found that COL17A1 plays an important role in maintaining epidermal stem cell activity and anti-aging (15, 37). PLLA maintained the low levels of COL17A1, a marker of basement membrane attachment, within EpiSCs compared to the control group (**Figures 6C, F**). The elevated expression of CK19 pairs in EpiSCs suggests a slow periodicity. As depicted in **Figures 6C** and **G**, the level of CK19 was reduced in the sixth generation of EpiSCs, while the PLLA+ fibroblast group demonstrated significantly heightened expression.

### 3.6 Mesotherapy of PLLA in rat skin

To test the effect of PLLA microspheres on the epidermis *in vivo*, an SD rat mesodermal injection model was established. PLLA microspheres (340mg/5mL) were intradermal injected on the left side of the rat’s back, while the right side was injected with an equal volume of saline (**Figures 2A, B**). Each small area was injected with 4 dots, and each dot was injected with a dose of 0.1mL.

As shown in **Figure 2C**, the normal injection depth is subdermal, whereas the injection site for mesotherapy is intradermal, in closer proximity to the epidermis, thereby facilitating better observation of PLLA’s impact on EpiSCs. Therefore, the PLLA group in our animal studies received mesoderm injections to assess their impact on the epidermis.

### 3.7 PLLA affects differentiation of rat EpiSCs *in vivo*

**Figure 7:**
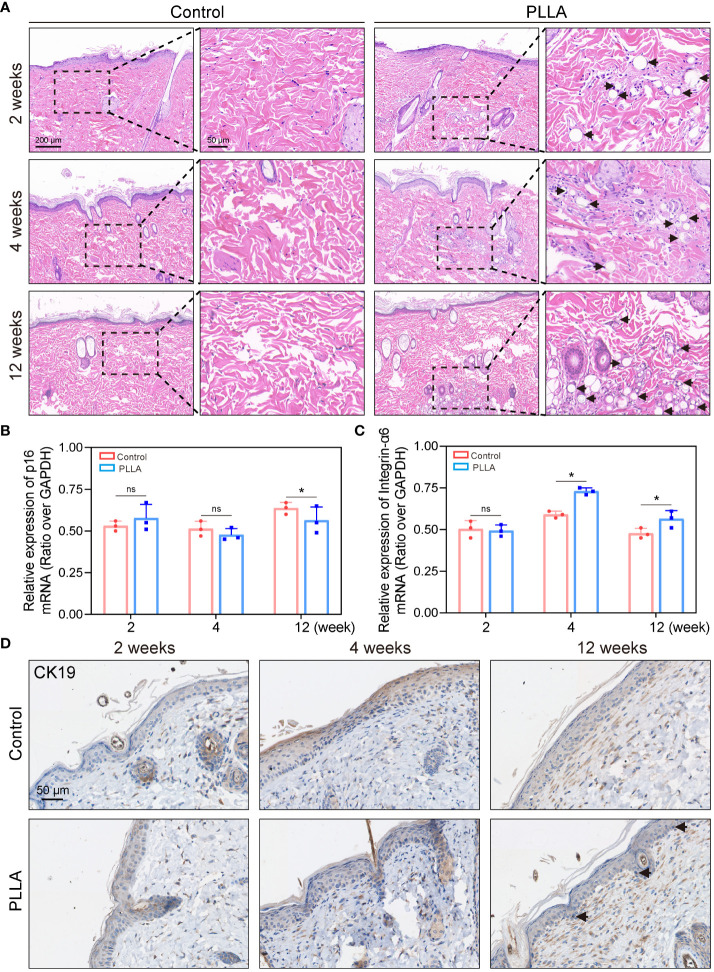
Effect of PLLA microspheres on rat epidermis *in vivo*. **(A)** Hematoxylin-eosin (H&E) Staining images of rat skin at 2,4 and 12 weeks after injection of PLLA microspheres. Q-PCR results of **(B)** p16 and **(C)** integrin-α6 in different treatment groups. **(D)** Immunohistochemical staining of CK19 in control group and PLLA group. Data are presented as mean ± SD. *indicates p < 0.05; ns means no significance.

The skin samples were collected from rats at 2, 4, and 12 weeks post-PLLA injection, followed by histological examination using H&E staining. It can be seen that the diameter of the PLLA microspheres in week 2 was larger, while the diameter of the microspheres in week 12 decreased as time progressed, suggesting that PLLA was degraded *in vivo* (**Figure 7A**). In addition, there were dense tissue structures and cells aggregation around the microspheres (**Figure 7A**). EpiSCs were extracted from the skin of rats at 2,4, and 12 weeks for q-PCR test. The q-PCR results revealed no significant difference in the p16 mRNA level between the two groups at both week 2 and week 4 (**Figure 7B**). While at the 12th week, p16 mRNA level in the PLLA injection group was observed to be lower than that in the control group, indicating a potential deceleration of rat EpiSCs aging by PLLA treatment (**Figure 7B**). The mRNA levels of integrin-α6 were higher in the PLLA group compared to the control group at both week 4 and week 12 (**Figure 7C**). The immunohistochemical staining results revealed no significant difference in CK19 expression during the initial 4-week period. However, at week 12, a distinct population of CK19-positive cells was observed in the basal cell layer of the epidermis in the PLLA group, suggesting that PLLA may induce differentiation of EpiSCs (**Figure 7D**).

## 4 Discussion

Skin aging, especially facial aging, is the consequence of common alterations in the soft and hard tissues of the body, face, and neck (1). The primary manifestations include thinning of facial skin, diminished elasticity, decreased moisture levels, appearance of wrinkles, loss of radiance and pigmentation; reduction in subcutaneous adipose tissue with localized accumulation (38, 39). Skin anti-aging and facial rejuvenation have garnered increasing attention in recent years (6, 40, 41). Herein, in this study, we assessed the anti-aging efficacy of intradermally injected PLLA microspheres on rat skin and conducted a preliminary investigation into the underlying mechanism.

Facial rejuvenation encompasses a range of treatments and approaches aimed at restoring the youthful appearance of an aging face, including both surgical and non-surgical interventions. Non-surgical methods have gained popularity due to their minimally invasive nature, such as botox injections, soft tissue fillers, laser or radiofrequency treatments, autologous fat grafting, and chemical peels, etc. (42–45) The durability of PLLA makes it a preferred choice as it requires less frequent refilling compared to other fillers and has a lifespan of two years or more (22, 43). The PLLA dermal filler is a biodegradable substance that can be injected to restore skin volume, smooth wrinkles, and enhance facial fullness (21). Unlike the immediate effect of hyaluronic acid fillers, the effect of PLLA is gradual (46). However, different PLLA products also have certain differences as the shapes are made into segments, or microspheres (47). Since the initial uneven particle size (20–100 μm) is prone to inflammatory reactions, the U.S. Food and Drug Administration (FDA) strictly controls the diameter of PLLA particles (25–40 μm) (24). PLLA microspheres are highly plastically and functional and controllable. subcutaneous injection of PLLA microspheres into the back skin of rabbits can strongly regenerate type I and III collagen for up to 13 months, and this is closely related to foreign body reaction (31). In terms of mechanism, immune cells, particularly macrophages, may exert a pivotal role in the anti-aging efficacy of PLLA (48, 49). PLLA could induce macrophage M2 polarization and collagen production by upregulating levels of IL-4, IL-13 and TGF-β in aging skin (23).

The skin is composed of two distinct layers, the epidermis and the dermis, which are interconnected by a delicate extracellular protein known as the basement membrane (50). Epidermal cells undergo continuous renewal, with new cells originating from both epidermal stem cells and other progenitor cells located deeper within the epidermis (9). Under normal circumstances, epidermal stem cells replicate and differentiate to generate fresh cells that replace damaged ones, thereby contributing to the maintenance of youthful skin (51). However, as one ages, these stem cells gradually diminish in number, resulting in a decline in regenerative capacity and subsequent aging of the skin (52). Collagen XVII (COL17A1), a crucial protein responsible for anchoring to the basement membrane, is situated within this specific region of the skin’s basement membrane and plays an essential role in preserving EpiSCs’ activity while safeguarding overall skin health (14). The loss of COL17A1 leads to reduced keratinocyte renewal and thinning of the epidermal layer—both being prominent morphological characteristics associated with age-related changes in the epidermis (15). The results of our study also demonstrated that PLLA maintained the expression of COL17A1 in the 6th generation EpiSCs, as compared to the control group (37).

Due to the protective effect of the epidermis, many treatments are not effectively targeted at the dermis; therefore, mesodermal therapy (intradermal injection) can effectively address this issue (53, 54). Currently, mesoderm therapy has been applied in the treatment of androgenic alopecia (55). Mesotherapy with HA-based fillers is also considered an effective method for restoring skin rejuvenation (56). The practice of PLLA mesoblastic injection, however, remains limited and its impact on the epidermis has yet to be investigated. We administered PLLA microspheres intradermally in rat back skin and observed a gradual degradation of the microspheres, which persisted for at least 12 weeks. The aging indexes of the PLLA group were superior to those of the control group at the 12th week, indicating that PLLA microspheres exhibited notable anti-aging effects.

*In vitro*, PLLA microspheres are mostly used as drug-loaded tools, and PLLA combined with other molecular materials can significantly promote the proliferation and collagen production of fibroblasts (57, 58). However, limited research has been conducted on the impact of PLLA alone on *in vitro* fibroblast. It was reported that PLLA could directly stimulate dermal fibroblasts to increase collagen I synthesis by activating p38, Akt and JNK signaling pathways (59). The injection of PLLA into the dermis, rather than the epidermis, results in a spatial separation between PLLA and the basement membrane of EpiSCs. Fibroblasts may establish a relationship with EpiSCs by secreting exosomes (60). Therefore, we established an *in vitro* co-culture system of EpiSCs and fibroblasts. Indeed, in the co-culture system, the anti-aging effect of PLLA and its ability to delay differentiation were more pronounced, indicating a significant role of fibroblasts in facilitating an “cross-talk” between PLLA and epidermal stem cells. It is worth noting that this interaction between fibroblasts and epidermal stem cells may also be reciprocal, with PLLA acting as a mediator. Further investigation is required to explore these dynamics.

## 5 Conclusion

In summary, our study is the first to investigate the impact of PLLA microspheres on the biological functionality of epidermal cells, with a specific focus on cellular senescence levels. *In vitro* experiments revealed that a concentration of 500μg/mL of PLLA microspheres promoted the proliferation and migration of primary rat EpiSCs, while also delaying their senescence and differentiation processes, thereby preserving their stemness. Similarly, *in vivo* mesodermal injection of PLLA yielded consistent results. These findings provide novel mechanistic insights into the anti-aging properties of PLLA for skin rejuvenation.

## Data availability statement

The original contributions presented in the study are included in the article/supplementary material. Further inquiries can be directed to the corresponding authors.

## Ethics statement

The animal study was approved by The Animal Ethics Committee of Guangdong Second Provincial General Hospital. The study was conducted in accordance with the local legislation and institutional requirements.

## Author contributions

YD: Conceptualization, Data curation, Funding acquisition, Methodology, Writing – original draft. YoZ: Conceptualization, Writing – original draft. HY: Writing – original draft, Data curation, Methodology. LZ: Data curation, Methodology, Formal analysis, Writing – review & editing. YaZ: Writing – review & editing. HW: Writing – review & editing, Project administration. ZH: Writing – review & editing, Conceptualization. SL: Conceptualization, Funding acquisition, Project administration, Writing – review & editing.

## References

[1] Liang Y Su W Wang F. Skin ageing: A progressive, multi-factorial condition demanding an integrated, multilayer-targeted remedy. Clin Cosmet Investig Dermatol. (2023) 16:1215–29. doi: 10.2147/CCID.S408765 PMC10182820 37192990

[2] Kohl E Steinbauer J Landthaler M Szeimies RM. Skin ageing. J Eur Acad Dermatol Venereol. (2011) 25:873–84. doi: 10.1111/j.1468-3083.2010.03963.x 21261751

[3] Csekes E Rackova L. Skin aging, cellular senescence and natural polyphenols. Int J Mol Sci. (2021) 22:12641. doi: 10.3390/ijms222312641 34884444 PMC8657738

[4] Mendelson B Wong CH. Changes in the facial skeleton with aging: implications and clinical applications in facial rejuvenation. Aesthet Plast Surg. (2020) 44:1151–8. doi: 10.1007/s00266-020-01823-x 32844267

[5] Gruber F Kremslehner C Eckhart L Tschachler E. Cell aging and cellular senescence in skin aging - Recent advances in fibroblast and keratinocyte biology. Exp Gerontol. (2020) 130:110780. doi: 10.1016/j.exger.2019.110780 31794850

[6] Quan T. Molecular insights of human skin epidermal and dermal aging. J Dermatol Sci. (2023) 112:48–53. doi: 10.1016/j.jdermsci.2023.08.006 37661473 PMC13155249

[7] Persa OD Koester J Niessen CM. Regulation of cell polarity and tissue architecture in epidermal aging and cancer. J Invest Dermatol. (2021) 141:1017–23. doi: 10.1016/j.jid.2020.12.012 33531135

[8] Kohler F Rodriguez-Paredes M. DNA methylation in epidermal differentiation, aging, and cancer. J Invest Dermatol. (2020) 140:38–47. doi: 10.1016/j.jid.2019.05.011 31427190

[9] Negri VA Watt FM. Understanding human epidermal stem cells at single-cell resolution. J Invest Dermatol. (2022) 142:2061–7. doi: 10.1016/j.jid.2022.04.003 PMC9826868 35570025

[10] Jackson CJ Tonseth KA Utheim TP. Cultured epidermal stem cells in regenerative medicine. Stem Cell Res Ther. (2017) 8:155. doi: 10.1186/s13287-017-0587-1 28676094 PMC5496160

[11] Dekoninck S Blanpain C. Stem cell dynamics, migration and plasticity during wound healing. Nat Cell Biol. (2019) 21:18–24. doi: 10.1038/s41556-018-0237-6 30602767 PMC7615151

[12] Hu W Zhang X Sheng H Liu Z Chen Z Huang Y. The mutual regulation between gammadelta T cells and macrophages during wound healing. J Leukoc Biol. (2023) 115(5):840–51. doi: 10.1093/jleuko/qiad087 37493223

[13] Jeong S Yoon S Kim S Jung J Kor M Shin K. Anti-wrinkle benefits of peptides complex stimulating skin basement membrane proteins expression. Int J Mol Sci. (2019) 21:73. doi: 10.3390/ijms21010073 31861912 PMC6981886

[14] Nanba D Toki F Asakawa K Matsumura H Shiraishi K Sayama K. EGFR-mediated epidermal stem cell motility drives skin regeneration through COL17A1 proteolysis. J Cell Biol. (2021) 220:e202012073. doi: 10.1083/jcb.202012073 34550317 PMC8563287

[15] Matsumura H Mohri Y Binh NT Morinaga H Fukuda M Ito M. Hair follicle aging is driven by transepidermal elimination of stem cells via COL17A1 proteolysis. Science. (2016) 351:aad4395. doi: 10.1126/science.aad4395 26912707

[16] Jiang C Javed A Kaiser L Nava MM Xu R Brandt DT. Mechanochemical control of epidermal stem cell divisions by B-plexins. Nat Commun. (2021) 12:1308. doi: 10.1038/s41467-021-21513-9 33637728 PMC7910479

[17] Bass LS. Injectable filler techniques for facial rejuvenation, volumization, and augmentation. Facial Plast Surg Clin North Am. (2015) 23:479–88. doi: 10.1016/j.fsc.2015.07.004 26505544

[18] Bukhari SNA Roswandi NL Waqas M Habib H Hussian F Khan S. Hyaluronic acid, a promising skin rejuvenating biomedicine: A review of recent updates and pre-clinical and clinical investigations on cosmetic and nutricosmetic effects. Int J Biol Macromol. (2018) 120:1682–95. doi: 10.1016/j.ijbiomac.2018.09.188 30287361

[19] Sundaram H Signorini M Liew S Almeida ART Wu Y Braz AV. Global aesthetics consensus: botulinum toxin type A–evidence-based review, emerging concepts, and consensus recommendations for aesthetic use, including updates on complications. Plast Reconstr Surg. (2016) 137:518e–29e. doi: 10.1097/01.prs.0000475758.63709.23 PMC5242214 26910696

[20] Breithaupt A Fitzgerald R. Collagen stimulators: poly-L-lactic acid and calcium hydroxyl apatite. Facial Plast Surg Clin North Am. (2015) 23:459–69. doi: 10.1016/j.fsc.2015.07.007 26505542

[21] Jabbar A Arruda S Sadick N. Off face usage of poly-L-lactic acid for body rejuvenation. J Drugs Dermatol. (2017) 16:489–94. 28628686

[22] Schierle CF Casas LA. Nonsurgical rejuvenation of the aging face with injectable poly-L-lactic acid for restoration of soft tissue volume. Aesthet Surg J. (2011) 31:95–109. doi: 10.1177/1090820X10391213 21239677

[23] Oh S Lee JH Kim HM Batsukh S Sung MJ Lim TH. Poly-L-lactic acid fillers improved dermal collagen synthesis by modulating M2 macrophage polarization in aged animal skin. Cells. (2023) 12:1320. doi: 10.3390/cells12091320 37174720 PMC10177436

[24] Christen MO. Collagen stimulators in body applications: A review focused on poly-L-lactic acid (PLLA). Clin Cosmet Investig Dermatol. (2022) 15:997–1019. doi: 10.2147/CCID.S359813 PMC9233565 35761856

[25] Avelar L Ong A Ong D Wai ACS Wai AYT Sungkyu J. Consensus recommendations on the use of injectable poly-l-lactic acid in Asian patients. J Cosmet Dermatol. (2023) 22:3223–31. doi: 10.1111/jocd.15969 37786340

[26] Catano JC. Mesotherapy-associated cutaneous infection. Am J Med Sci. (2019) 357:e21–2. doi: 10.1016/j.amjms.2019.02.015 30926084

[27] Markiewicz-Tomczyk A Budzisz E. Erkiert-polguj A. A subjective and objective assessment of combined methods of applying chemical peels and microneedling in antiaging treatments. J Clin Med. (2023) 12 1869. doi: 10.3390/jcm12051869 36902657 PMC10003688

[28] Hu L Zhao K Song WM. Effect of mesotherapy with nanochip in the treatment of facial rejuvenation. J Cosmet Laser Ther. (2020) 22:84–9. doi: 10.1080/14764172.2020.1740272 32223473

[29] Li X Guo L Chen J Liang H Liu Y Chen W. Intravenous injection of human umbilical cord-derived mesenchymal stem cells ameliorates not only blood glucose but also nephrotic complication of diabetic rats through autophagy-mediated anti-senescent mechanism. Stem Cell Res Ther. (2023) 14:146. doi: 10.1186/s13287-023-03354-z 37248536 PMC10228071

[30] Agle KA Vongsa RA Dwinell MB. Chemokine stimulation promotes enterocyte migration through laminin-specific integrins. Am J Physiol Gastrointest Liver Physiol. (2011) 301:G968–980. doi: 10.1152/ajpgi.00208.2011 PMC3233784 21921288

[31] Zhang Y Liang H Luo Q Chen J Zhao N Gao w. *In vivo* inducing collagen regeneration of biodegradable polymer microspheres. Regener Biomater. (2021) 8:rbab042. doi: 10.1093/rb/rbab042 PMC8364987 34408912

[32] Huang S Hu Z Wang P Zhang Y Cao X Dong Y. Rat epidermal stem cells promote the angiogenesis of full-thickness wounds. Stem Cell Res Ther. (2020) 11:344. doi: 10.1186/s13287-020-01844-y 32771044 PMC7414990

[33] Xu H Yang H Wang Z Tang Q Cao X Chen C. Epidermal stem cell derived exosomes alleviate excessive autophagy induced endothelial cell apoptosis by delivering miR200b-3p to diabetic wounds. J Invest Dermatol. (2023) 144:1134–47.e2. doi: 10.1016/j.jid.2023.08.030 37838331

[34] Kato T Liu N Morinaga H Asakawa K Muraguchi T Muroyama Y. Dynamic stem cell selection safeguards the genomic integrity of the epidermis. Dev Cell. (2021) 56:3309–20 e3305. doi: 10.1016/j.devcel.2021.11.018 34932948

[35] Chen S Takahara M Kido M Takeuchi S Uchi H Tu Y. Increased expression of an epidermal stem cell marker, cytokeratin 19, in cutaneous squamous cell carcinoma. Br J Dermatol. (2008) 159:952–5. doi: 10.1111/bjd.2008.159.issue-4 18647309

[36] Quist SR Eckardt M Kriesche A Gollnick HP. Expression of epidermal stem cell markers in skin and adnexal Malignancies. Br J Dermatol. (2016) 175:520–30. doi: 10.1111/bjd.14494 26914519

[37] Liu N Matsumura H Kato T Ichinose S Takada A Namiki T. Stem cell competition orchestrates skin homeostasis and ageing. Nature. (2019) 568:344–50. doi: 10.1038/s41586-019-1085-7 30944469

[38] Ansary TM Hossain MR Kamiya K Komine M Ohtsuki M. Inflammatory molecules associated with ultraviolet radiation-mediated skin aging. Int J Mol Sci. (2021) 22:3974. doi: 10.3390/ijms22083974 33921444 PMC8069861

[39] Pils V Ring N Valdivieso K Lämmermann I Gruber F Schosserer M. Promises and challenges of senolytics in skin regeneration, pathology and ageing. Mech Ageing Dev. (2021) 200:111588. doi: 10.1016/j.mad.2021.111588 34678388

[40] Ahmed IA Mikail MA Zamakshshari N Abdullah AH. Natural anti-aging skincare: role and potential. Biogerontology. (2020) 21:293–310. doi: 10.1007/s10522-020-09865-z 32162126

[41] He X Wan F Su W Xie W. Research progress on skin aging and active ingredients. Molecules. (2023) 28:5556. doi: 10.3390/molecules28145556 37513428 PMC10385838

[42] Borba A Matayoshi S Rodrigues M. Avoiding complications on the upper face treatment with botulinum toxin: A practical guide. Aesthet Plast Surg. (2022) 46:385–94. doi: 10.1007/s00266-021-02483-1 PMC8328485 34341857

[43] Sarubi J Avelar LET Nero MPD Kamamoto C Morais M. Facial rejuvenation on the use of injectable poly-L-lactic acid and hyaluronic acid: Combined technique. J Cosmet Dermatol. (2022) 21:5261–3. doi: 10.1111/jocd.14902 35253957

[44] Crowley JS Kream E Fabi S Cohen SR. Facial rejuvenation with fat grafting and fillers. Aesthet Surg J. (2021) 41:S31–8. doi: 10.1093/asj/sjab014 34002771

[45] Thomas JR. Update on facial skin rejuvenation technology. Facial Plast Surg Clin North Am. (2020) 28:xi. doi: 10.1016/j.fsc.2019.10.001 31779947

[46] Palm M Weinkle S Cho Y LaTowsky B Prather H. A randomized study on PLLA using higher dilution volume and immediate use following reconstitution. J Drugs Dermatol. (2021) 20:760–6. doi: 10.36849/JDD.6034 34232000

[47] Fitzgerald R Bass LM Goldberg DJ Graivier MH Lorenc ZP. Physiochemical characteristics of poly-L-lactic acid (PLLA). Aesthet Surg J. (2018) 38:S13–7. doi: 10.1093/asj/sjy012 29897517

[48] Sureshchandra S Messaoudi I. Aging and macrophages: Not standing the test of time? J Leukoc Biol. (2022) 112:1369–70. doi: 10.1002/JLB.3CE0322-145R PMC10321356 35766198

[49] Pang J Koh TJ. Proliferation of monocytes and macrophages in homeostasis, infection, injury, and disease. J Leukoc Biol. (2023) 114:532–46. doi: 10.1093/jleuko/qiad093 PMC10673715 37555460

[50] Jeong S Yoon S Kim S Jung J Kor M Shin K. Anti-wrinkle benefits of peptides complex stimulating skin basement membrane proteins expression. Int J Mol Sci. (2019) 21:73. doi: 10.3390/ijms21010073 31861912 PMC6981886

[51] Singh R. Basal cells in the epidermis and epidermal differentiation. Stem Cell Rev Rep. (2022) 18:1883–91. doi: 10.1007/s12015-021-10256-1 35080747

[52] Mokry J Pisal R. Development and maintenance of epidermal stem cells in skin adnexa. Int J Mol Sci. (2020) 21:9736. doi: 10.3390/ijms21249736 33419358 PMC7766199

[53] Plachouri KM Georgiou S. Mesotherapy: Safety profile and management of complications. J Cosmet Dermatol. (2019) 18:1601–5. doi: 10.1111/jocd.13115 31444843

[54] Lee JC Daniels MA Roth MZ. Mesotherapy, microneedling, and chemical peels. Clin Plast Surg. (2016) 43:583–95. doi: 10.1016/j.cps.2016.03.004 27363773

[55] Saceda-Corralo D Moustafa F Moreno-Arrones O Jaen-Olasolo P Vano-Galvan S Camacho F. Mesotherapy with dutasteride for androgenetic alopecia: A retrospective study in real clinical practice. J Drugs Dermatol. (2022) 21:742–7. doi: 10.36849/JDD.6610 35816059

[56] Iranmanesh B Khalili M Mohammadi S Amiri R Aflatoonian M. Employing hyaluronic acid-based mesotherapy for facial rejuvenation. J Cosmet Dermatol. (2022) 21:6605–18. doi: 10.1111/jocd.15341 36098653

[57] Das R Le TT Schiff B Chorsi MT Park J Lam P. Biodegradable piezoelectric skin-wound scaffold. Biomaterials. (2023) 301:122270. doi: 10.1016/j.biomaterials.2023.122270 37591188 PMC10528909

[58] Lu H Oh HH Kawazoe N Yamagishi K Chen G. PLLA-collagen and PLLA-gelatin hybrid scaffolds with funnel-like porous structure for skin tissue engineering. Sci Technol Adv Mater. (2012) 13:064210. doi: 10.1088/1468-6996/13/6/064210 27877537 PMC5099770

[59] Kim SA Kim HS Jung JW Suh SI Ryoo YW. Poly-L-lactic acid increases collagen gene expression and synthesis in cultured dermal fibroblast (Hs68) through the p38 MAPK pathway. Ann Dermatol. (2019) 31:97–100. doi: 10.5021/ad.2019.31.1.97 33911550 PMC7992693

[60] Wu JY Wu SN Zhang LP Zhao XS Li Y Yang OY. Stem cell-derived exosomes: A new method for reversing skin aging. Tissue Eng Regener Med. (2022) 19:961–8. doi: 10.1007/s13770-022-00461-5 PMC9477989 35809187

